# Identification of fungal enzymes involving 3-phenoxybenzoic acid degradation by using enzymes inhibitors and inducers

**DOI:** 10.1016/j.mex.2019.100772

**Published:** 2019-12-17

**Authors:** Jiayuan Zhao, Xiaofeng Chen, Dongying Jia, Kai Yao

**Affiliations:** a College of Life Science, Sichuan Normal University, 610101, Chengdu, Sichuan, PR China; b College of Biomass Science and Engineering, Sichuan University, 610065, Chengdu, Sichuan, PR China

**Keywords:** Identification of fungal enzymes involving 3-phenoxybenzoic acid degradation by using enzymes inhibitors and inducers, 3-phenoxybenzoic acid, Degrading-enzymes, Enzyme inhibitor, Enzyme inducer

## Abstract

Pyrethroid residues in food and the environment can be bio-transformed into 3-phenoxybenzoic acid (3-PBA); It is more toxic than the parent compounds, and has been detected in milk, soil, and human urine. In this study, when incubated at 30 °C and 180 rpm for 48 h, mycelial pellets during logarithmic growth phase were obtained and washed 2 times by phosphate buffer. The cell debris solutions and filter liquor from inducible and non-inducible samples were cultured with 3-PBA and its intermediate metabolites at same condition, and the location and induction of enzymes were analyzed by the degradation. Then Cytochrome P450 (CYP450), lignin peroxidase (LiP), laccase, manganese peroxidase (MnP), and dioxygenase were selected as candidate enzymes due to these oxidases existing in the fungi and capable of degrading the contaminants with similar structures of these compounds, and CuSO_4_, NaN_3_, AgNO_3_, EDTA or piperonyl butoxide (PBO) were used as the enzymes inhibitors and inducers. The degradation of 3-PBA and its intermediate metabolites and the fungal biomass in presence of enzymes inhibitors and inducers was arranged to analyze the possible degrading-enzymes, and the co-metabolic enzymes and pathways can be reasoned. This study provided a promising method for studying the co-metabolic enzymes of 3-PBA degradation by fungi.

• The presented MethodsX was conducted for co-metabolic enzymes and pathways of 3-PBA degradation.
• The culturing condition for presenting enzyme properties were investigated.
• The candidate enzymes were analyzing based on location, induction of enzymes, fungal enzyme systems and chemical structures of these compounds.

## Graphical abstract

**Figure:**
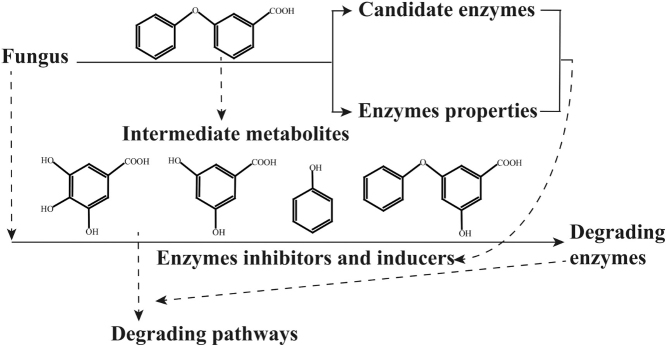

**Specification Table**

**Table:** 

Subject Area:	Environmental Science
More specific subject area:	Biodegradation
Method name:	Identification of fungal enzymes involving 3-phenoxybenzoic acid degradation by using enzymes inhibitors and inducers
Name and reference of original method:	The measurement for 3-phenoxybenzoic acid and its intermediate metabolites in medium by high-performance liquid chromatography (HPLC) was based on the methods reported by Zhao et al. (2019) [1] and Zhao et al. (2016) [2]. The inducible or non-inducible nature of degrading-enzymes was measured as the protocol reported by Fang et al. (2015) [3]. The activity of Cytochrome P450 (CYP450) was measured using previously described by Uno et al. (2017) [4]; the activity of lignin peroxidase (LiP), laccase, and manganese peroxidase (MnP) was determined according to Guo et al. (2014) [5]; Dioxygenase activity was measured according to Sari et al. (2012) [6]. The inhibitors and inducers of CYP 450, LiP, laccase, MnP, and dioxygenase were selected based on the results reported by Ikehata et al. (2004) [7], Oszajca et al. (2016) [8], Sari et al. (2012) [6], Senthivelan et al. (2016) [9] and Yeom and Sligar (1997) [10]. [1]. Zhao, J. Y., et al., 2019. Co-metabolic enzymes and pathways of 3-phenoxybenzoic acid degradation by Aspergillus oryzae M-4. Ecotoxicology and Environmental Safety. x, x-x. https://doi.org/10.1016/j.ecoenv.2019.109953 [2]. Zhao, J. Y., et al., 2016. Co-metabolic degradation of beta-cypermethrin and 3-phenoxybenzoic acid by co-culture of Bacillus licheniformis B-1 and Aspergillus oryzae M-4. Plos One. 11, 14. https://doi.org/10.1371/journal.pone.0166796 [3]. Fang, S. M., et al., 2015. Enzymatic degradation of aliphatic nitriles by Rhodococcus rhodochrous BX2, a versatile nitrile-degrading bacterium. Bioresource Technology. 185, 28-34. https://doi.org/10.1016/j.biortech.2015.02.078 [4]. Uno, T., et al., 2017. Functional characterization of CYP52G3 from Aspergillus oryzae and its application for bioconversion and synthesis of hydroxyl flavanone and steroids. Biotechnology and Applied Biochemistry. 64, 385-391. https://doi.org/10.1002/bab.1496 [5]. Guo, D. Z., et al., 2014. A comparative study on the degradation of gallic acid by Aspergillus oryzae and Phanerochaete chrysosporium. Water Science and Technology. 70, 175-181. https://doi.org/10.2166/wst.2014.213 [6]. Sari, A. A., et al., 2012. Determination of co-metabolism for 1,1,1-trichloro-2,2-bis(4-chlorophenyl) ethane (DDT) degradation with enzymes from Trametes versicolor U97. Journal of Bioscience and Bioengineering. 114, 176-181. https://doi.org/10.1016/j.jbiosc.2012.03.006 [7]. Ikehata, K., et al., 2004. Recent developments in the production of extracellular fungal peroxidases and laccases for waste treatment. Journal of Environmental Engineering and Science. 3, 1-19. https://doi.org/10.1139/s03-077 [8]. Oszajca, M., et al., 2016. Mechanistic studies on versatile metal-assisted hydrogen peroxide activation processes for biomedical and environmental incentives. Coordination Chemistry Reviews. 327, 143-165. https://doi.org/10.1016/j.ccr.2016.05.013 [9]. Senthivelan, T., et al., 2016. Recent trends in fungal laccase for various industrial applications: an eco-friendly approach - a review. Biotechnology and Bioprocess Engineering. 21, 19-38. https://doi.org/10.1007/s12257-015-0278-7 [10]. Yeom, H. Y., Sligar, S. G., 1997. Oxygen activation by cytochrome P450(BM-3): Effects of mutating an active site acidic residue. Archives of Biochemistry and Biophysics. 337, 209-216. https://doi.org/10.1006/abbi.1996.9763
Resource availability:	N/A

## Method details

### Introduction

Chemical pesticides are important components of modern global agricultural systems, leading to a substantial improvement in crop yields by controlling insects [1]. Nevertheless, some negative influence including disastrous contamination of ecosystems (i.e., dust, soils, air, sediments, and water) and adulterating human foods have been appeared due to the extensive application of chemical pesticides [1]. Since the partial or total ban on organochlorine and organophosphorus pesticides, pyrethroids have been widely used and account for 25 % of the global pesticides market [2]. Long term and excessive spraying have become common methods for pyrethroids application. The pyrethroid residues in the environment and food as a result of these spraying methods has attracted more attention because of their potential to contaminate the food supply chain and threaten human health [3]. As an intermediate metabolite of pyrethroids, 3-phenoxybenzoic acid (3-PBA) is more toxic than its parent compounds and has been detected in milk, soil, and human urine [[3], [4], [5]]. 3-PBA can be metabolized to less potent metabolites through microbial degradation. It has been predicted that oxidases were the main enzymes involving 3-PBA degradation by microorganisms [[4], [5], [6]], and the degrading-enzymes needed further investigation.

Some contaminants containing similar chemical structures with 3-PBA and its intermediate metabolites could be degraded by oxidases such as laccase, MnP, LiP, and dioxygenase [7,8], and many fungi including Aspergillus oryzae have these enzyme genes [9]. Therefore, it could be reasoned that these oxidases might also participate the degradation.

### Method

**Fig. 1:**
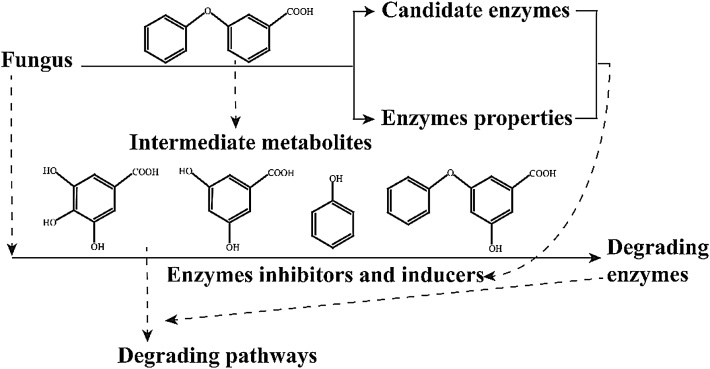
Procedure to study the co-metabolic enzymes and pathways of 3-PBA degradation by fungi.

This MethodsX is presented to researchers as a step by step procedure to study the co-metabolic enzymes and pathways of 3-PBA degradation by fungi, as shown in Fig. 1. Generally, the metabolites of 3-PBA degradation could be identified by gas chromatography-mass spectrometry [10,11]. The strain *Aspergillus oryzae* M-4 that is able to degrade 3-PBA via co-metabolism was obtained from the soy sauce koji. The intermediate metabolites of 3-PBA degradation include 3-hydroxy-5-phenoxy benzoic acid, phenol, gallic acid, and catechol [12,13]. The concentrations of 3-PBA and its intermediate metabolites can be detected by high-performance liquid chromatography (HPLC) following the methods reported by Zhao et al. (2016 and 2019) [12,13]. Degradation of 3-PBA and its intermediate metabolites was calculated according to the following equation [12,13]:

(1) Degradation (%) = (1‒ C_k_ / C) × 100

where C_k_ and C represent the residual compound concentration (mM) and the initial compound concentration (mM) in the sample solution, respectively.

The location of enzymes, the inducible or non-inducible nature of enzymes, and the chemical structures of intermediate metabolites are important characters for analyzing the candidates.

The location and induction of enzymes involving 3-PBA and intermediate metabolites were investigated. The degrading-strain was serially cultured in media containing 3-PBA and its intermediate metabolites (0.24 mM), which served as the induced sample. The medium without any additives served as the non-inducible sample. Each of the replicate samples were cultured at 30 °C and 180 rpm for different duration (24, 36, 48, 60, and 72 h). The fungal biomass was measured according to Zhao et al. (2019) [12]. The fungal biomasses were varying with different intermediate metabolites. At 48 h, strain M-4 was in logarithmic growth phase in all the samples. The culture for subsequent experiments were obtained in logarithmic phase growth. A 30 mL culture was filtered through a paper filter, and the mycelial pellets and filter liquor were collected. Mycelial pellets were washed 1–5 times, and the concentrations of 3-PBA and its intermediate metabolites in mycelial pellets were measured each wash. Their concentrations were almost zero after washing them twice. Then, mycelial pellets were resuspended in 30 mL of pH 7.0 phosphate buffer (50 mM). The cell debris solution was prepared after ultrasonication of mycelial pellets following the method reported by Fang et al. (2015) [14].

The same volumes of filter liquor and cell debris solutions were added into phosphate buffer solution containing 0.048 mM each of 3-PBA, and intermediate metabolites and the degradation of the compounds was measured. The reaction was conducted in different duration (24, 48, 72 h) at 30 °C and 180 rpm. Then, the reaction was terminated by adding equal volumes of acetonitrile. The concentrations of 3-PBA or its potential metabolites were measured, and degradation was calculated. The results indicated that degradation of 3-PBA and its potential metabolites by the filter liquor and cell debris solutions at 72 h was not significantly higher than that at 48 h. The location of enzymes involving 3-PBA and intermediate metabolites were understood.

Based on the chemical structures of 3-PBA and intermediate metabolites and fungal enzyme system, the candidate enzymes could be speculated. Then, compared the enzymes activities in the treatments with and without 3-PBA and analyzed the degrading-enzymes. One and half mL of the inoculum of degrading-strain was added to a 250 mL Erlenmeyer flask containing 30 mL of medium with 0.24 mM 3-PBA. The concentrations of 3-PBA and its potential metabolites were measured after incubation at 180 rpm and 30 °C for 24, 48, 72 and 96 h, respectively. The sample with the greatest shift in concentrations was selected. The cultures of degrading-strain were filtered through a paper filter, and both the mycelial pellets and filter liquor were collected to determine the activity of the following enzymes. Cytochrome P450 (CYP450) activity was measured using previously described methods [15]. The activity of lignin peroxidase (LiP), laccase, and manganese peroxidase (MnP) was determined according to Guo et al. (2014) [9]. Dioxygenase activity was measured according to Sari et al. (2012) [16]. The candidate enzymes could be speculated by the location of enzymes, the inducible or non-inducible nature of enzymes, and the chemical structures of intermediate metabolites.

In order to identify the enzymes involving 3-PBA or its intermediate metabolites from the candidate enzymes, the degradation of these compounds was studied in presence of the inhibitors and inducers of these enzymes. The inhibitors and inducers of CYP450, LiP, laccase, MnP, and dioxygenase were selected according to previous reporters [[17], [18], [19], [20]]. Some solutions of enzymes inhibitors were prepared. CuSO_4_, NaN_3_, and AgNO_3_ were dissolved in water, piperonyl butoxide (PBO) dissolved in EtOH, and EDTA was dissolved in NaOH solution (10 %, w/v). The degradation was proceeded in five 250-mL flasks containing 30 mL of MD medium with 0.24 mM 3-PBA or its intermediate metabolites, respectively. Then, different volumes of ethyl alcohol, CuSO_4_, NaN_3_, AgNO_3_, or PBO filter-sterilized solutions were added, and their final concentrations were 0.1, 0.5, 1.0, 5.0 and 10.0 0.24 mM, respectively. After that, the1.5 mL inoculum was added and incubated at 30 °C on a shaker at 180 rpm for 72 h. After 72 h, the concentrations of 3-PBA and its intermediate metabolites in the culture were detected by HPLC. The degradation of 3-PBA and its intermediate metabolites was calculated according to Eq. (1). The biomass of strain M-4 (Dry weight, g/L) was measured according to Zhao et al. (2019). The degrading-enzymes of 3-PBA or its intermediate metabolites were understood by analyzing the relationship between degradation and enzymes inhibitors and inducers.

### Conclusion

In this study, a new method for investigating the degradation enzymes of 3-PBA and its intermediate metabolites was present. The candidate enzymes were analyzed based on the chemical structure of 3-PBA and its intermediate metabolites and the reported property of fungi. The location and induction of enzymes were used to evaluate the candidate enzymes, and predict the enzymes inhibitors and inducers. Further, the effects of inhibitors and inducers on degradation of 3-PBA and intermediate metabolites were used to analyzed the degrading-enzymes. These procedures will be a helpful guide for researchers to analyze the co-metabolic enzymes of 3-PBA degradation by fungi
